# Diffusion Tensor Imaging for Evaluating Biliary Atresia in Infants and Neonates

**DOI:** 10.1371/journal.pone.0168477

**Published:** 2016-12-19

**Authors:** Bo Liu, Jinhua Cai, Jin Zhu, Helin Zheng, Yun Zhang, Longlun Wang

**Affiliations:** 1 Department of Radiology, Children’s Hospital of Chongqing Medical University, Chongqing, China; 2 Department of Pathology, Children’s Hospital of Chongqing Medical University, Chongqing, China; Northwestern University Feinberg School of Medicine, UNITED STATES

## Abstract

**Background:**

Preliminary studies have shown that diffusion tensor imaging (DTI) is helpful in evaluating liver disorders. However, there is no published literature on the use of DTI in the diagnosis of biliary atresia (BA). This study aimed to investigate the diagnostic value of the liver average apparent diffusion coefficient (ADC) and fractional anisotropy (FA) measured using DTI for BA in neonates and infants.

**Methods:**

Fifty-nine patients with infant jaundice were included in this study. DTI was performed with b factors of 0 and 1000 s/mm^2^. Liver fibrosis in the BA group was determined and graded (F0, F1, F2, F3, F4) based on the pathological findings. Statistical analyses were performed to determine the diagnostic accuracy of DTI for BA.

**Results:**

The ADC value was significantly lower in the BA group [(1.262±0.127)×10^−3^ mm^2^/s] than in the non-BA group [(1.430±0.149)×10^−3^ mm^2^/s, (*P*<0.001)]. The area under the receiver operating characteristic curve was 0.805±0.058 (*P*<0.001) for ADC. With a cut-off value of 1.317×10^−3^ mm^2^/s, ADC achieved a sensitivity of 75% and a specificity of 81.5% for the differential diagnosis of BA and non-BA. In the BA group, the ADC value was significantly correlated with fibrotic stage. Further analysis showed that the ADC value of stage F0 was significantly higher than that of stages F1, F2, F3 and F4, whereas there were no significant differences among stages F1, F2, F3 and F4.

**Conclusion:**

Hepatic ADC measured with DTI can be used as an adjunct to other noninvasive imaging methods in the differential diagnosis of BA and non-BA. ADC was helpful in detecting liver fibrosis but not in differentiating the fibrotic grades.

## Introduction

Biliary atresia (BA) is a progressive disorder that can result in hepatic fibrosis and cirrhosis [1]. This condition is unique to neonates and infants, and its etiology remains unknown. If left untreated, BA can lead to death, usually during infancy. Early diagnosis improves the prognosis for BA but remains challenging [2–5]. At present, the diagnostic methods for BA are divided into invasive and noninvasive categories. Among invasive methods, which include surgical exploration (SE), laparoscopic exploration (LE) and liver biopsy, intraoperative cholangiography (IC) is often considered the diagnostic gold standard for BA because it allows a visual assessment of the biliary tree [5]. Among noninvasive methods, hepatobiliary scintigraphy uses radiation, and the diagnostic accuracy of ultrasonography or color Doppler ultrasonography needs to be improved. The recently reported Fibroscan (transient elastography) can be used to assess liver fibrosis in BA patients but must be further validated using a large sample size [6]. Other noninvasive imaging techniques, including computed tomography and magnetic resonance imaging (MRI), have been used to predict the risk of esophageal variceal bleeding in BA [7, 8]. MRI has also been used to differentiate BA from total parenteral nutrition-associated cholestasis by using the indices of lobar difference and left lateral hepatic angle [9]. In a previous study, we applied three-dimensional magnetic resonance cholangiopancreatography (3D-MRCP) to diagnose BA, and the results showed high sensitivity but low specificity, with unsatisfactory accuracy [10].

In recent years, diffusion-weighted imaging (DWI) has become an important noninvasive technique for evaluating ultrastructural changes in liver tissue, especially in chronic liver disease. The usefulness of DWI when using an MR imaging system for the detection and assessment of liver fibrosis has been reported in several studies, which revealed a decrease in the apparent diffusion coefficient (ADC) in cirrhotic liver tissue [11–18]. Mesude T, et al. [19] found that the hepatic ADC value decreased significantly in BA compared to the controls and suggested that the right hepatic ADC measured with DWI by 1.5-T MRI could be useful for the diagnosis and long-term follow-up of cirrhotic severity in BA patients. Compared with DWI, diffusion tensor imaging (DTI) uses additional gradients in multiple dimensions and has potential advantages in the detection of fibrosis because different diffusion directions are calculated [20–22]. Tosun M, et al [23] evaluated liver fibrosis using DTI. The study verified the importance of DTI for assessing liver fibrosis using multiple indices such as ADC and fractional anisotropy (FA) values. However, there is no literature on the application of DTI in the diagnosis and evaluation of BA.

In this study, DTI was performed in patients with clinical suspicion of BA, and the average ADC and FA were measured and compared between the BA and non-BA groups. Our aim was to investigate the value of DTI for the differential diagnosis and assessment of liver fibrosis in BA, and we expected to find a relatively reliable and noninvasive method for the detection and assessment of BA.

## Materials and Methods

### Ethics statement

The study protocol was approved by the Human Ethics Committee of the Children’s Hospital of Chongqing Medical University. Written informed consent was obtained from the parents or guardians of all patients before the examinations.

### Patients

Fifty-nine patients (32 male, 27 female; mean age, 79.6±48.1 days; age range, 20 days to 272 days) with infant jaundice and clinical suspicion of BA were included in our study between January 2012 and April 2014. The inclusion criterion was presentation with more than one clinical symptom, such as jaundice, pale-colored stools, hepatomegaly, and/or dark urine. The exclusion criteria included abandoning treatment, indefinite diagnosis, diagnosis as non-BA by 3D-MRCP, or follow-up of less than one year. Blood biochemistry tests were obtained 2 days before surgery or MRI. For MRI examination, chloral hydrate was orally administrated at a dose of 50 mg/kg for sedation.

### Imaging data collection and post-processing

This study was performed with a 1.5-T MRI unit (Signa Propeller HD; GE Medical Systems, Milwaukee, WI, USA) using a single-channel quadrature head coil. Routine axial T1- and T2-weighted imaging were performed first. Then, the 3D-MRCP images were obtained as described in detail previously [10]. Finally, the axial-plane DTI was performed using a single-shot spin echo echo-planar-imaging (EPI) sequence with the following parameters: TR 6000 msec, TE 95.7 msec, matrix 128×128, b factors of 0 and 1000 s/mm^2^, slice thickness 5.0 mm, slice gap 0 mm, number of excitations 2, sampling bandwidth 250 KHz and sensitive diffusion gradient field applied in 15 directions. The total imaging time of DTI was three minutes and 24 seconds, and the field of view covered the diaphragmatic dome and margo interior hepatic.

**Fig 1:**
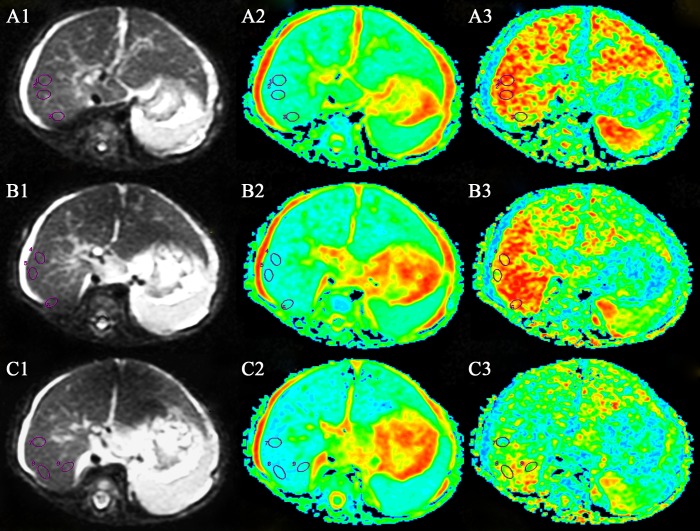
Measurement of the apparent diffusion coefficient and fractional anisotropy on diffusion tensor imaging reconstructed images. A 165-day-old female infant with biliary atresia. Three regions of interest were drawn on each original image (b = 0), and three consecutive images (A1, B1 and C1) above the hepatic porta were included. The mean ADC values were obtained from the nine total ROIs on the ADC map (A2, B2 and C2), and the mean FA values were obtained from the FA maps (A3, B3 and C3).

Using the Functool post-processing software on a workstation (ADW4.4; GE Medical Systems, Milwaukee, WI, USA), FA and ADC maps were automatically calculated from the original DTI data. The regions of interest (ROIs) were placed on the right hepatic lobe on the b = 0 images, with an effort to avoid interference from the surrounding abdominal wall, vascular and biliary structures, and then transferred automatically to the parametric maps where the ADC and FA values were calculated. Three ROIs were drawn on each image, and three consecutive images above the hepatic porta were included. The mean value of the nine total ROIs was recorded as the final ADC or FA for each patient (Fig 1). The results of the above procedures were interpreted in consensus by two pediatric radiologists (with eight and ten years of experience in abdominal MRI, respectively) who were blinded to the clinical information.

### Histopathological evaluation

The patients with clinical and MRI suspicion of BA underwent SE, IC, LE or pathological examination (PE). PE was performed with a mean delay of 3 days (range 1–7 days) after MRI examination. All specimens were evaluated by an experienced pathologist. Fibrosis of the liver tissue was classified histopathologically in accordance with the METAVIR scoring system, which uses a 5-point scale for fibrotic stages (F0: no fibrosis; F1: fibrous portal expansion; F2: fibrous portal expansion, few fibrotic septae; F3: numerous fibrotic septae, no cirrhosis; F4: cirrhosis) [24].

### Statistical analysis

All patients were divided into the BA group and non-BA group by SE, IC, LE, PE or clinical outcome. The PE results were used as the reference when evaluating the fibrotic stages. Statistical analyses were performed using Statistical Package for the Social Scences 18.0 software (IBM Corporation, Armonk, NY, USA). For the quantitative evaluation of DTI, the FA and ADC values were compared. The fitness of numeric data set to a normal distribution and homogeneity of variance were determined using both the Kolmogorov-Smirnov test and Levene test. To compare the differences in age, blood test results, ROI, ADC and FA between the BA and non-BA groups, the independent sample *t* test was applied. We used the Chi-square test to analyze the gender difference between the BA and non-BA groups. *P*<0.05 was considered statistically significant. In the BA group, the difference in ADC or FA between fibrotic stages was compared using one-way ANOVA and the least significant difference (LSD)-*t* test. To evaluate the diagnostic competence of ADC for the detection of BA and describe the sensitivity and specificity of the test, receiver operating characteristic (ROC) analysis was performed.

## Results

### Surgical and therapy results

Thirty-two patients were diagnosed as BA, including two type I, one type II, 27 type III and two type III with common bile duct cyst. Twenty-seven patients were diagnosed as non-BA, of whom five had cytomegalovirus hepatitis, five had biliary stenosis, six had common bile duct cyst, nine had infant hepatitis (IH), one had biliary ductal distention and one had cholestasis. Thirty-two BA patients underwent PE, and the fibrotic stages were determined.

### Comparison between the BA and non-BA groups

There was no statistically significant difference in age, gender, blood test results or area of ROIs between the BA and non-BA groups **(**Table 1**)**. The ADC value in the BA group was significantly lower than that in the non-BA group (*P*<0.001), whereas the FA value was not significantly different between the BA and non-BA groups (*P* = 0.093) (Table 2).

**Table 1:** Demographic characteristics, blood test results and ROI between the BA and non-BA groups (n = 59).

	BA (n = 32)	non-BA (n = 27)	*t* or *χ*^*2*^	*P* value
**Age (days)**	85.3±54.0	72.9±40.1	*t* = 0.983	0.330
**Gender (M/F)**	16/16	16/11	*χ*^*2*^ = 0.506	0.477
**Blood test**				
**AST (IU/L)**	297.13±117.703	291.37±103.427	*t* = 0.198	0.844
**ALT (IU/L)**	184.63±87.946	161.74±69.563	*t* = 1.093	0.279
**TBIL (μmol/L)**	175.25±59.333	158.04±43.142	*t* = 1.253	0.215
**DBIL (μmol/L)**	75.25±37.560	85.07±38.191	*t* = -0.993	0.325
**TBA (μmol/L)**	109.63±53.452	129.52±45.914	*t* = -1.518	0.135
**ROI(mm**^**2**^**)**	66.93±13.437	72.17±16.128	*t* = -1.360	0.179

BA, biliary atresia; M/F, male/female; AST, aspartate aminotransferase; ALT, alanine aminotransferase; TBIL, total bilirubin; DBIL, direct bilirubin; TBA, total bile acid; ROI, region of interest.

**Table 2:** Comparison of ADC or FA values between the BA and non-BA groups (n = 59).

	BA group (n = 32)	non-BA group (n = 27)	*t*	*P*
**ADC (×10**^**−3**^ **mm**^**2**^**/s)**	1.262±0.127	1.430±0.149	-4.666	*<*0.001
**FA**	0.335±0.068	0.361±0.044	-1.711	0.093

BA, biliary atresia; ADC, apparent diffusion coefficient; FA, fractional anisotropy.

### Correlation between ADC or FA and fibrotic stages in the BA group

The ADC values showed a decreasing trend, whereas the FA values showed an increasing trend with increasing fibrotic stage (F0-F4). There were statistically significant differences between the ADC values (*P* = 0.017) but not between the FA values (*P* = 0.183) at different fibrotic stages (Table 3).

**Table 3:** Correlation of ADC or FA with fibrotic stages in the BA group (n = 32).

	Fibrotic stages						
	F0(n = 2)	F1 (n = 14)	F2 (n = 4)	F3 (n = 7)	F4 (n = 5)	F	*P*
**ADC (×10**^**−3 mm2**^**/s)**	1.490±0.066	1.295±0.123	1.232±0.095	1.204±0.093	1.184±0.108	3.644	0.017
**FA**	0.289±0.064	0.314±0.054	0.331±0.067	0.353±0.051	0.391±0.107	1.682	0.183

ADC, apparent diffusion coefficient; FA, fractional anisotropy. F0, F1, F2, F3 and F4 represent different fibrotic stages (F0: no fibrosis; F1: fibrous portal expansion; F2: fibrous portal expansion, few fibrotic septae; F3: numerous fibrotic septae, no cirrhosis; F4: cirrhosis).

Further LSD analysis showed that the ADC value of stage F0 was significantly higher than that of stages F1, F2, F3 and F4, whereas there were no significant differences among stages F1, F2, F3 and F4 (Table 4).

**Table 4:** The least significant difference (LSD) of the ADC (×10^−3^ mm^2^/s) between fibrotic stages in the BA group (n = 32).

	F0	F1	F2	F3	F4
**F0**	N/A	*P* = 0.027	*P* = 0.012	*P* = 0.003	*P* = 0.003
**F1**	*P* = 0.027	N/A	*	*	*
**F2**	*P* = 0.012	*	N/A	*	*
**F3**	*P* = 0.003	*	*	N/A	*
**F4**	*P* = 0.003	*	*	*	N/A

ADC, apparent diffusion coefficient; FA, fractional anisotropy; LSD, least significant difference. F0, F1, F2, F3 and F4 represent different fibrotic stages (F0: no fibrosis; F1: fibrous portal expansion; F2: fibrous portal expansion, few fibrotic septae; F3: numerous fibrotic septae, no cirrhosis; F4: cirrhosis); N/A represents not applicable.

* represents *P*>0.05.

### Analysis of ROC drawn by combining the BA and non-BA groups

**Fig 2:**
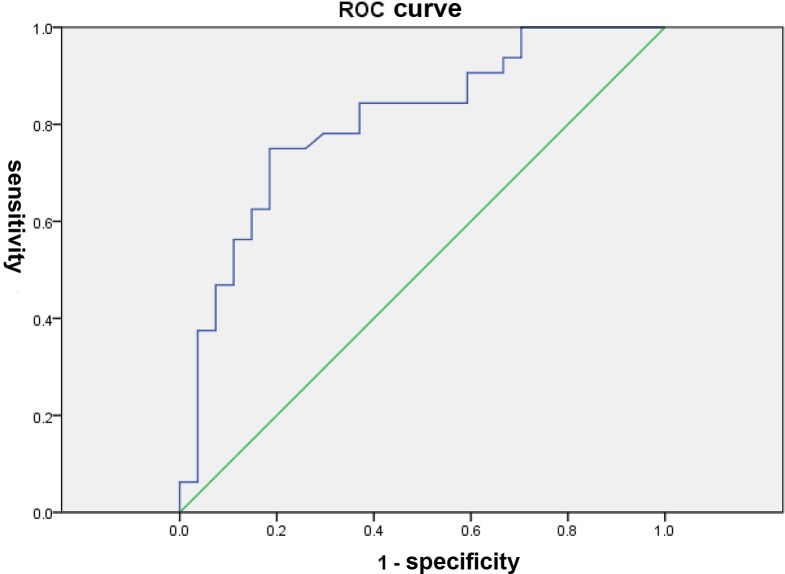
Receiver operating characteristic curve of the average apparent diffusion coefficient for the detection of biliary atresia. The area under the ROC curve was 0.805±0.058 (*P*<0.001) for ADC. With a cut-off value of 1.317×10^−3^ mm^2^/s, ADC reached a sensitivity of 75% and a specificity of 81.5% for the differential diagnosis of BA and non-BA.

The area under the ROC curve was 0.805±0.058 (*P*<0.001) for ADC. With a cut-off value of 1.317×10^−3^ mm^2^/s for ADC, DTI reached a sensitivity of 75% and a specificity of 81.5% for the differential diagnosis of BA and non-BA (Fig 2).

## Discussion

This study showed that the ADC value measured with DTI is significantly lower in BA and could be helpful in both the diagnosis of BA and the detection of liver fibrosis in infants and neonates. In addition, at a cut-off value of 1.317×10^−3^ mm^2^/s, ADC reached a sensitivity of 75% and a specificity of 81.5% for the differential diagnosis of BA and non-BA. Our previous research indicates that the diagnostic sensitivity of 3D-MRCP for BA is 99.04% but that its specificity is only 36.05% [10]. Therefore, DTI combined with 3D-MRCP may provide a solution for the diagnosis of BA, and this possibility should be further validated.

A few recent reports have suggested that DWI is helpful in the pathological evaluation of diffusive lesions of the adult liver and that the ADC of cirrhotic liver tissue is lower than that of normal liver tissue [11–18]. Some scholars have found that ADC and FA are potentially valuable in detecting liver fibrosis at early stages and for monitoring its progression, as demonstrated by scanning the livers of rats with DTI by 1.5-T MRI [25]. The results of our study are similar to the results of this previous study. The ADC value was significantly lower in the BA group. This finding suggests that fibrotic biliary ducts and cirrhotic liver tissue could easily appear in the course of BA progression and thus may influence the diffusion of water in the liver. Liver fibrosis is a nonspecific response to chronic liver disease and leads to excess synthesis of the extracellular matrix, especially collagen fibers [26–28]. Thus, the presence of collagen fibers restricts diffusion in the fibrotic liver, and the ADC values are decreased in cirrhotic liver compared with normal liver [23]. By contrast, there was no significant difference in the FA value between the BA group and the non-BA group, possibly due to the microstructural features of liver fibrosis. The fibrosis in the liver occurred along the interlobular septum and was distributed in a disordered form without a homogeneous direction. Such a distribution of fibrosis did not influence the FA value, which reflects the heterogeneity of the molecules in different directions.

In the present study, the ADC values showed a decreasing trend as the degree of liver fibrosis in BA patients increased (F0-F4). There was a statistically significant difference in ADC among fibrotic stages (F0, F1, F2, F3, and F4). Further statistical analysis by LSD demonstrated significant differences in ADC only between F0 and the other stages, whereas no significant differences were detected among stages F1, F2, F3 and F4. This finding suggests that ADC can be used to roughly determine the presence or absence of fibrosis in BA but can not evaluate the stages of liver fibrosis. A previous study reported that the ADC values measured by 3-T MRI were correlated with liver fibrotic stages, particularly in distinguishing high- from low-grade fibrosis [23]. This study showed that the application of a higher magnetic field MRI system could detect fibrotic stages with greater sensitivity. In addition, the small sample size used in our study could influence the results of the differences among different fibrotic stages. Further study with a large number of cases is needed to validate the effectiveness of ADC in distinguishing fibrotic stages.

In this study, our aim was to evaluate the use of DTI in the differentiation of BA and other disorders whose clinical manifestations were similar to BA. For this purpose, we did not include normal children in the non-BA group for comparison because normal children who did not display clinical symptoms can be easily excluded by clinical physical examinations and would thus not influence the differential diagnosis of BA and non-BA. Furthermore, because our previous research showed that the negative predictive value of 3D-MRCP for BA was 96.88% [10], children who were diagnosed as normal or non-BA by 3D-MRCP were excluded from the study. Under these conditions, our results showed that ADC was significantly lower in the BA group than in the non-BA group, suggesting that the fibrosis in BA could be more serious or more frequent than in other disorders. This suggestion was verified by pathological findings, which showed various degrees of fibrosis in most BA cases but severe fibrosis in only one of the 12 non-BA cases that underwent PE.

The drawing method for ROIs, including the location and size, could potentially influence the results. To avoid this bias as much as possible, in this study, the ROIs were placed on the right hepatic lobe rather than the left lobe for the following reasons. First, hepatic fibrosis involves the left lobe less frequently than the right lobe, as confirmed by pathological studies [29,30]. Second, susceptibility artifacts are more frequently found in the left hepatic regions [19], which can lead to measurement errors. Third, according to recent reports, the measurement reproducibility and reliability of left hepatic lobar ADC values is lower than that of the right ADC in adults and BA patients [31, 32]. In addition, we drew 3 ROIs on each image for 3 consecutive images and calculated the mean value of 9 ROIs. The statistical analysis showed no difference in the size of the ROIs between the BA group and the non-BA group. This analysis further reduced the bias of the measurement of the ADC and FA value.

The b factor in DTI is important and should be chosen according to the study purpose. In this study, we performed DTI with b factors of 0 and 1000 s/mm^2^ because a larger b value indicates a lower contribution of perfusion to the ADC value [30, 31]. Free-breathing was also used in the DTI in this study as recommended for its good reproducibility and shorter acquisition time compared with multiple breath-holding, respiratory-triggered, and navigator-triggered techniques [33] and because it is especially suitable for infants and neonates.

One limitation of our study is that the potential confounding effect of hepatic steatosis on ADC or FA values could not be excluded. Recently, several studies have raised concerns about the relationship between liver fat and ADC values [34–36]. Although the results were not in consensus, all researchers indicated that ADC values should be cautiously interpreted in the presence of liver steatosis. In our study, unfortunately, we did not quantify the liver fat content and analyze its correlation with ADC or FA values, because PE results were not available for patients in the control group. In addition, an accurate quantification of liver steatosis was impossible due to the relatively small amount of the sample obtained by biopsy. In future studies, an animal model of biliary atresia should be used to explore the exact impact of hepatic steatosis on the ADC or FA values.

In conclusion, hepatic ADC measured with DTI may be useful for the differential diagnosis of BA and non-BA in neonates and infants and may aid the detection of liver fibrosis. The sensitivity and specificity of ADC for BA diagnosis should be further improved and combined with other noninvasive imaging methods.

## Supporting Information

S1 Table Raw data of all patients (n = 59). This table shows the original data of all patients, including age, gender, areas of ROI, apparent diffusion coefficient and fractional anisotropy.
(SAV)

S2 Table Raw data of the patients in the BA group (n = 32). This table includes the original data of apparent diffusion coefficient and fractional anisotropy among different fibrotic stages in the BA group.
(SAV)
